# Roles of pyroptosis and immune infiltration in aortic dissection

**DOI:** 10.3389/fmolb.2024.1277818

**Published:** 2024-03-19

**Authors:** Xiaogang Ge, Qiqi Cai, Yangyang Cai, Caiguo Mou, Junhui Fu, Feng Lin

**Affiliations:** ^1^ Vascular and Endovascular Surgery, Huangyan Hospital Affiliated to Wenzhou Medical University, Taizhou First People’s Hospital, Taizhou, Zhejiang, China; ^2^ Department of Emergency Intensive Care Unit, Huangyan Hospital Affiliated to Wenzhou Medical University, Taizhou First People’s Hospital, Taizhou, Zhejiang, China

**Keywords:** aortic dissection, bioinformatics, pyroptosis, immune infiltration, database

## Abstract

**Introduction:** Aortic dissection (AD) is often fatal, and its pathogenesis involves immune infiltration and pyroptosis, though the molecular pathways connecting these processes remain unclear. This study aimed to investigate the role of immune infiltration and pyroptosis in AD pathogenesis using bioinformatics analysis.

**Methods:** Two Gene Expression Omnibus datasets and a Gene Cards dataset of pyroptosis-related genes (PRGs) were utilized. Immunological infiltration was assessed using CIBERSORT, and AD diagnostic markers were identified through univariate logistic regression and least absolute shrinkage and selection operator regression. Interaction networks were constructed using STRING, and weighted gene correlation network analysis (WGCNA) was employed to identify important modules and essential genes. Single-sample gene set enrichment analysis determined immune infiltration, and Pearson correlation analysis assessed the association of key genes with infiltrating immune cells.

**Results:** Thirty-one PRGs associated with inflammatory response, vascular epidermal growth factor receptor, and Rap1 signaling pathways were identified. WGCNA revealed seven important genes within a critical module. CIBERSORT detected immune cell infiltration, indicating significant changes in immune cell infiltration and pyroptosis genes in AD and their connections.

**Discussion:** Our findings suggest that key PRGs may serve as indicators for AD or high-risk individuals. Understanding the role of pyroptosis and immune cell infiltration in AD pathogenesis may lead to the development of novel molecular-targeted therapies for AD.

**Conclusion:** This study provides insights into the molecular mechanisms underlying AD pathogenesis, highlighting the importance of immune infiltration and pyroptosis. Identification of diagnostic markers and potential therapeutic targets may improve the management of AD and reduce associated morbidity and mortality.

## 1 Introduction

Aortic dissection (AD) is a fatal condition for which there is currently no reliable drug treatment (Yang et al., 2020). Various risk factors for AD have been identified, including hypertension, atherosclerosis, and hypercholesterolemia (Hibino et al., 2022). In addition, smoking, drug abuse (especially cocaine), pregnancy, history of cardiac surgery, history of AD, aortic constriction, aortic aneurysm, and aortic mitral valve are other factors known to predispose an individual to AD (Parve et al., 2017). Typical AD is characterized by an endoluminal flap that divides the actual lumen from the fake lumen (Bossone et al., 2018). The Stanford approach distinguishes between two types of AD, types A and B, based on the location and degree of the trapping. Type A entrapment involves the ascending aorta (and may spread to the descending aorta), whereas type B entrapment exclusively affects the descending aorta. This classification is essential to determine the appropriate treatment strategy (Clouse et al., 2004). In the United States and Europe, the prevalence of AD ranges between 3.5 and 7.2 per 100,000 persons (Smedberg et al., 2020). The incidence of AD has shown a continuous increase in recent years, with a 50% and 30% increase in the annual incidence of AD reported for men and women, respectively (Olsson et al., 2006). Although the in-hospital mortality rate for AD has decreased significantly from 31% to 22% and the overall 5-year survival rate increased from 5% to 32%, the median survival of patients with AD is still only 3 days (Clouse et al., 2004; Bossone and Eagle, 2021). The high mortality rate of AD cannot be disregarded; thus, further research into the underlying factors affecting the condition will assist in our comprehension of its pathogenic processes, guiding clinical diagnosis and treatment and improving clinical prognosis.

Immune infiltration, as a key immune system response, is crucial in both the formation and remodeling phases of AD. Immune infiltration has been observed within the arterial wall of AD, and leukocyte cell adhesion, monocyte migration, and myeloid leukocyte migration are significantly increased in AD (Li et al., 2022). The monocyte-macrophage system plays a critical role in the immunological inflammatory response during the development of AD (Gao et al., 2021). Neutrophil infiltration is currently considered to predominate in the acute phase of the disease (Yoshida et al., 2019), whereas macrophages appear after 1 day and peak at 2–7 days (Xu and Burke, 2013). In an immune-high group of patients with AD, the percentages of infiltrating CD8^+^ T cells and M1-type macrophages were found to be considerably higher than those in the immune-low group (Li et al., 2022). Substantial lymphoplasmacytic infiltration has also been reported in AD (Uchida et al., 2018). Activated T cells and macrophages may enhance smooth muscle cell elimination (He et al., 2006), increased vascular inflammation (Lian et al., 2019), increased elastic fiber and extracellular matrix degradation (Gomez et al., 2016), and vascular smooth muscle cell death (Wu et al., 2022). These processes eventually lead to aortic dilatation and rupture. In contrast, immunological responses modulate AD arterial wall remodeling, which involves complicated interactions between cells and immune inflammatory factors. As a result, we suspect that immune infiltration contributes significantly to the onset of AD through controlling inflammation as well as the deterioration and remodeling of the artery walls.

Recent evidence has also pointed to a role of pyroptosis, a form of inflammatory cell death, in the development of AD (Zhang X. et al., 2020). AD occurs when inflammatory vesicles are activated in response to a stimulatory danger signal (Duan et al., 2020), leading to the release of pro-inflammatory factors to ultimately induce pyroptosis (Sun et al., 2021). Therefore, inhibition of the pyroptosis pathway offers a possible strategy to rescue AD (Duan et al., 2020). Pyroptosis is common in immune cells such macrophages, monocytes, and dendritic cells (Taabazuing et al., 2017). Pyroptotic cells are considered to be more inflammatory and immunogenic than apoptotic cells (Tsuchiya et al., 2019) because they release cellular contents such as inflammatory cytokines and damage-associated molecular patterns (Ruan et al., 2018). However, there is a lack of research on the potential correlations between pyroptosis and immune infiltration in AD.

**FIGURE 1:**
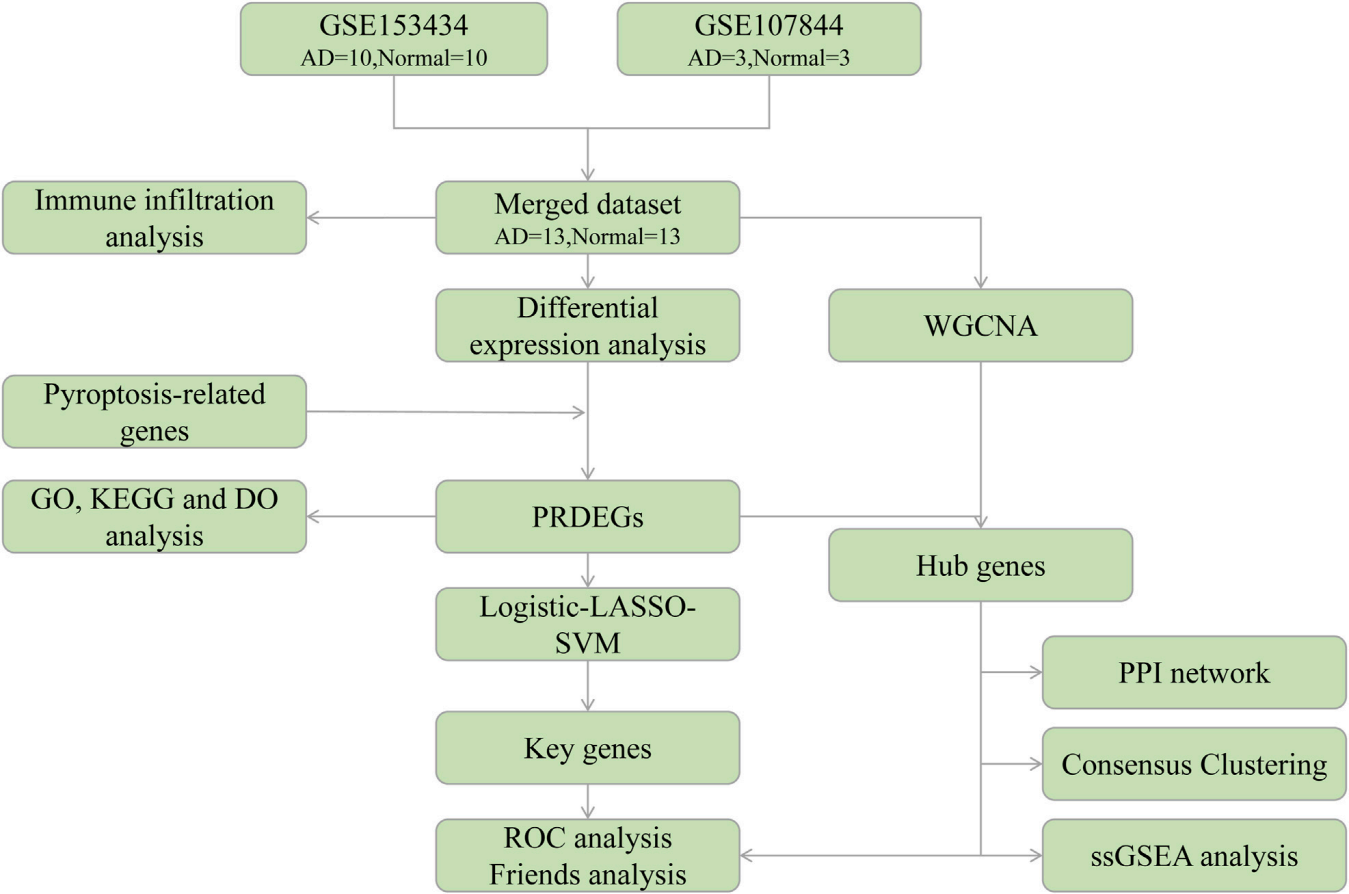
Analysis flow chart. AD, aortic dissection; PRDEGs, pyroptosis-related differentially expressed genes; WGCNA, weighted gene correlation network analysis; PPI, protein-protein interaction network; LASSO, least absolute shrinkage and selection operator; SVM, support vector machine; GO, Gene Ontology; KEGG, Kyoto Encyclopedia of Genes and Genomes; DO, Disease Ontology; ROC, receiver operating characteristic; ssGSEA, single-sample gene set enrichment analysis.

To explore these associations, in this study, we used bioinformatics to analyze the Gene Expression Omnibus (GEO) database of genes associated with AD. We combined these datasets with a pyroptosis-related genes (PRGs) dataset to determine the probable functional processes and essential genes of pyroptosis. The link between PRG hub genes and invading immune cells was further investigated to elucidate the roles of pyroptosis and immunological processes during AD progression. A flow chart of the overall analysis process is provided in Figure 1.

## 2 Materials and methods

### 2.1 Data acquisition and pre-processing

**FIGURE 2:**
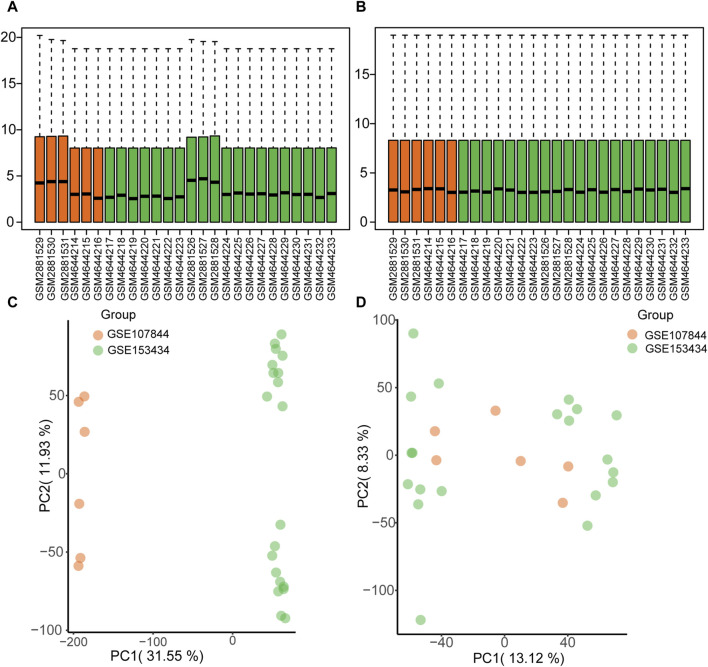
Gene expression distribution before and after batch correction for the GSE153434 and GSE107844 datasets. **(A)** Box plots of gene expression distribution before normalization. **(B)** Box plots of gene expression distribution after normalization. **(C)** Principal components analysis (PCA) before batch correction. **(D)** PCA analysis after batch correction.

The GSE1534343 (AD = 10, control = 10) and GSE107844 (AD = 3, control = 3) gene expression profile datasets were obtained from the GEO database (Barrett et al., 2007) using the R program GEOquery (Davis and Meltzer, 2007). The species source of both datasets is human (*Homo sapiens*); the GSE153434 dataset was compiled from the HiSeq X Ten microarray sequencing platform (platform number GPL20795), and the tissue source is the ascending aorta, whereas the dataset GSE107844 was derived from the Illumina chip platform (platform number GPL20301), and the tissue source is the thoracic aorta. Details of the two datasets are provided in Table 1. For each dataset, platform annotation information was downloaded to convert probe names to gene names, and multiple expression results for specific genes were replaced with the mean of the expression values. The data were normalized using the normalizeBetweenArrays function (Further details about this function can be found at: https://web.mit.edu/∼r/current/arch/i386_linux26/lib/R/library/limma/html/05Normalization.html) in the limma package and merged with batch correction using the Combat function with the R package sva23 (More information about the Combat function can be found at: https://rdrr.io/bioc/sva/man/ComBat.html). After pre-processing and merging, the two datasets showed a similar distribution of gene expression values (Figures 2A, B), and the batch effects of the two datasets were corrected (Figures 2C, D). Finally, the GSE153434 and GSE107844 datasets were merged and used as the combined dataset for further analysis.

**TABLE 1:** Dataset information.

	GSE153434	GSE107844
Platform	GPL20795	GPL20301
Species	*Homo sapiens*	*Homo sapiens*
Tissue	ascending aorta	thoracic aorta
Samples in AD group	10	3
Samples in Normal group	10	43
Reference	Exaggerated Autophagy in Stanford Type A Aortic Dissection: A Transcriptome Pilot Analysis of Human Ascending Aortic Tissues	—

AD, aortae dissectione.

### 2.2 Differential gene expression analysis and acquisition of PRGs

The GeneCards database (http://www.genecards.org/) (Safran et al., 2010) incorporates gene-centric data from approximately 150 online sources, including genomic, transcriptional, protein biology, genetic, clinical, and functional data. By searching for “pyroptosis” in the GeneCards database, we obtained a total of 436 PRGs for subsequent analysis. Based on the grouping information in the combined dataset, we used the R package limma to obtain genes with differential expression between AD and controls according to log fold change (FC) > 1 (upregulated expression) or < −1 (downregulated expression) and adjusted *p*-value < 0.05.

### 2.3 Functional enrichment of PRGs

Gene Ontology (GO) enrichment (The gene ontology, 2019) is commonly used for large-scale functional enrichment studies of genes at various dimensions and levels, including biological process, molecular function, and cellular component (Ashburner et al., 2000). The Kyoto Encyclopedia of Genes and Genomes (KEGG) is a popular database containing data related to genomes, biological processes, diseases, and medications (Kanehisa and Goto, 2000). The R package “clusterprofiler” (Yu et al., 2012; Wu et al., 2021) was used to perform GO functional annotation, pathway, and disease enrichment analyses, and the “pathview” package was used to display the KEGG pathways with significant alterations in AD (Luo and Brouwer, 2013). GO terms and pathways with *p* < 0.05 were deemed to be significantly enriched.

### 2.4 Immune cell analysis

CIBERSORT (Newman et al., 2019) is a web-based tool on the R platform for deconvolution of an expression matrix of human immune cell subtypes based on the principle of linear support vector regression. According to a gene expression signature collection comprising 22 known immune cell subtypes, CIBERSORT can identify the infiltration status of immune cells in sequenced samples. The CIBERSORT program was used in this study to evaluate the status of infiltrating immune cells in the combined data set, and Spearman correlation analysis was employed to calculate the interrelationships between different immune cells.

### 2.5 Gene set enrichment analysis (GSEA)

GSEA is a computational enrichment method developed at the Broad Institute (Subramanian et al., 2005) with corresponding analysis software and a gene set database (MSigDB), which is widely used to determine whether a set of predefined genes differs significantly between two biological states and to estimate changes in pathway and biological process activity in samples of expression data sets. The reference gene set “c2.all.v2022.1.Hs.symbols.gmt” was downloaded from the MSigDB database (Liberzon et al., 2015) based on the gene expression profile dataset, which was then enriched and visualized using the GSEA method included in the R package “clusterProfiler” to investigate the differences in biological processes between the AD and control samples. The following settings were used in this GSEA: seed number = 2022, calculations = 1000, at least 10 genes contained in each gene set, and a maximum number of genes featured of 500. The *p*-value was adjusted using Benjamini–Hochberg correction. The screening parameters for substantial enrichment were *p* < 0.05 and false discovery rate (q.value) < 0.05.

### 2.6 Single-factor logistic regression, least absolute shrinkage and selection operator (LASSO), and support vector machine (SVM) analyses

We used univariate logistic regression analysis, LASSO regression analysis, and the SVM-RFE technique for feature selection to search for AD diagnostic indicators. Initially, we used univariate logistic regression analysis to determine the relationship between the expression level of each differentially expressed gene in patients with AD, retaining genes with a *p*-value < 0.05. Subsequently, we utilized the SVM and LASSO (Friedman et al., 2010) algorithms of the glmnet package to downscale and select significant variables in the one-way logistic regression analysis. To assess the diagnostic performance of the model, we used the R package “pROC” (Robin et al., 2011) to plot the receiver operating characteristic (ROC) curve and computed the area under the curve (AUC) values.

### 2.7 Weighted gene correlation network analysis (WGCNA)

WGCNA (Langfelder and Horvath, 2008) seeks to find co-expressed gene modules, investigate the link between gene networks and phenotypes, and investigate the network’s core genes. The pickSoftTreshold function calculates the soft threshold, with 5 being the optimal soft threshold. A scale-free network is then constructed based on the soft threshold, followed by construction of a topology matrix and hierarchical clustering. The gene modules were identified by dynamic cutting using 50 as the minimum number of genes in the module, and eigengenes were computed. Inter-module correlations were built using the module eigengenes and hierarchical clustering, and Pearson correlation analysis was used to evaluate the relationships between modules and between modules and clinical characteristics. To identify important genes associated with pyroptosis in AD, genes from the most relevant modules for AD were intersected with the differentially expressed PRGs.

### 2.8 Construction of interaction networks

The STRING database (Szklarczyk et al., 2019), comprising 9.6 million proteins and 138 million protein–protein interactions (PPIs) from 2031 species, is used for searching known proteins and predicting PPIs. STRING includes experimental data, the results of text mining of PubMed abstracts, a synthesis of other database data results, and outcomes anticipated using bioinformatics approaches. The STRING database was used to build PPI networks for intersecting gene sets of modular and differentially expressed PRGs linked with AD, which were then visualized using Cytoscape (v3.9.1) software (Shannon et al., 2003). We also utilized Cytoscape’s Molecular Complex Detection (MCODE) tool to cluster and identify the essential modules in the PPI network. The microRNAs (miRNAs) linked with the identified hub genes were retrieved and intersected using starBase version 3.0 to investigate the association between hub genes and miRNAs (Huang et al., 2020). Lastly, Cytoscape software was used to visualize the mRNA–miRNA regulation network.

### 2.9 Molecular fractionation

Consensus clustering (Lock and Dunson, 2013) is a resampling-based algorithm that can be used to identify each member of a cluster and its subgroup number and for verifying the reasonableness of the generated clusters. Consistent clustering involves multiple iterations over subsamples of the dataset, indicating cluster stability and parameter decisions by using subsampling to induce sampling variability. We used the R package “ConsensusClusterPlus” (Wilkerson and Hayes, 2010) to perform consistent clustering of the dataset using the key genes associated with pyroptosis in AD to facilitate better differentiation between different subtypes of AD samples. In this process, 80% of the total samples were drawn in 1000 repetitions with the following settings: clusterAlg = “hc” and distance = “pearson.”

### 2.10 Immune infiltration correlation analysis

The quantity of certain infiltrating immune cells and the activity of specific immune responses can be estimated using single-sample GSEA (ssGSEA). According to published data of tumor immune infiltration, ssGSEA identified 28 gene sets for identifying distinct tumor-infiltrating immune cell types, containing numerous human immune cell subtypes (e.g., CD8^+^ T cells, dendritic cells, macrophages, regulatory T cells). Among ssGSEA methods, the R package GSVA protocol can yield enrichment scores for assessing the infiltration of each immune cell type within each sample. Therefore, we used the “GSVA” R package for ssGSEA on the immune cell infiltration gene set obtained from the literature (Zhang B. et al., 2020). The correlation of key genes associated with AD with infiltrating immune cells was determined by Pearson correlation analysis. Heat maps were created in R using the “ggplot2” visualization tool.

### 2.11 Statistical analysis

All data computations and statistical analyses were carried out using R programming (version 4.2.0, available at https://www.r-project.org/). The statistical significance of normally distributed variables was evaluated using an independent Student’s t-test, whereas differences between non-normally distributed variables were investigated using the Mann–Whitney *U*-test. Statistical significance was judged with a two-sided *p*-value < 0.05.

## 3 Results

### 3.1 Differential gene expression and enrichment analysis

**FIGURE 3:**
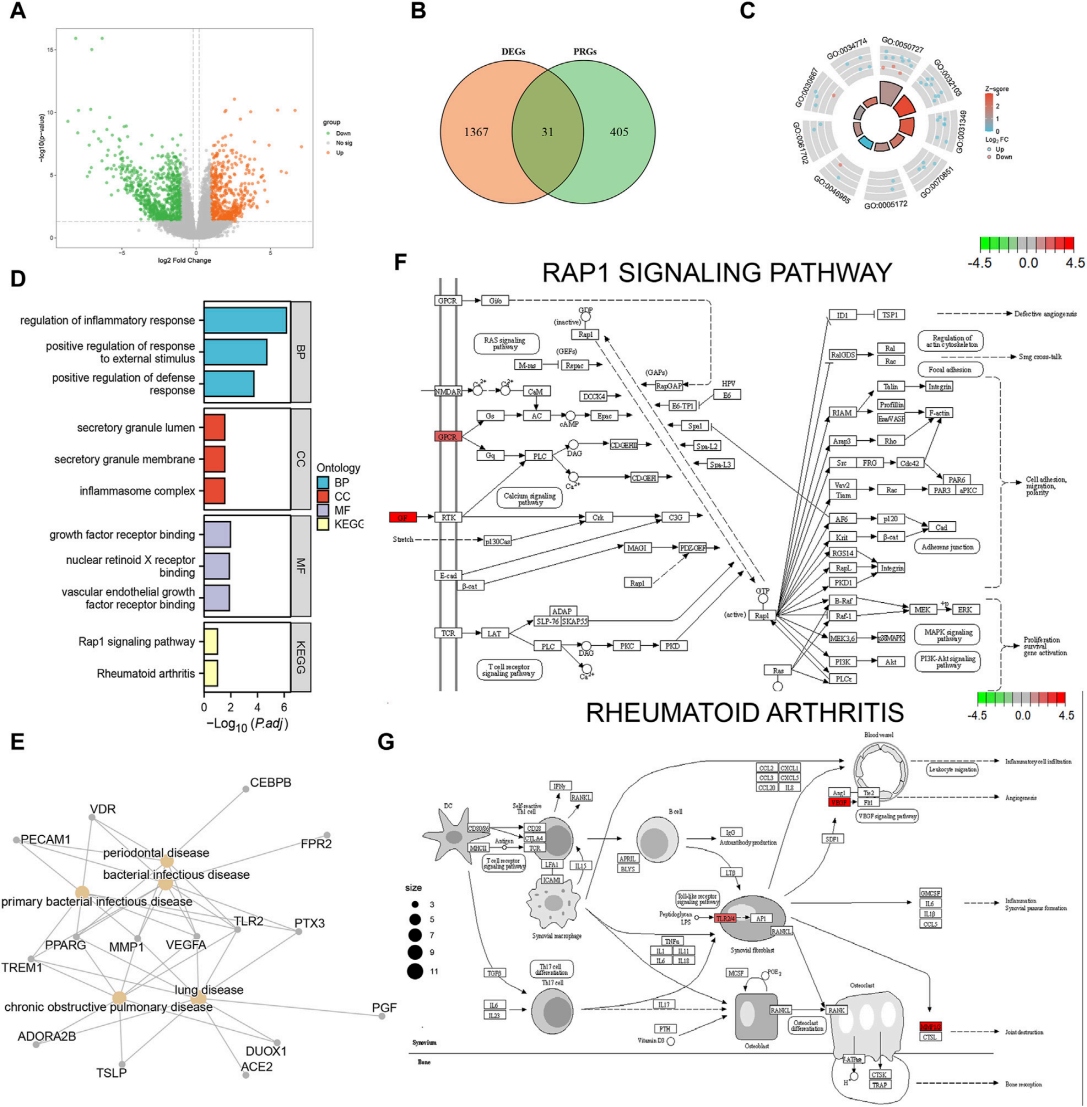
Functional enrichment analysis of differentially expressed pyroptosis-related genes. **(A)** Volcano plot display of the differential gene expression analysis from the combined aortic dissection (AD) dataset. **(B)** Differentially expressed pyroptosis-related genes; orange circles indicate differentially expressed genes (DEGs), green circles indicate pyroptosis-related genes (PRGs), and the intersection indicates differentially expressed pyroptosis-related genes in AD. **(C)** GO enrichment pathway circle diagram. The diagram can be divided into two parts: the inner circle and outer circle. Each bar in the inner circle corresponds to an entry, and the height is inversely related to the adjusted *p*-value (p.adj). The color of the corresponding filled bar represents the Z-score value corresponding to the entry. **(D)** Gene Ontology (GO) and Kyoto Encyclopedia of Genes and Genomes (KEGG) functional enrichment analysis; the horizontal coordinates are differentially expressed PRGs enriched to various entries and the vertical coordinate is the–log10 (*p*-value). Light blue indicates biological processes, red indicates cellular components, purple indicates molecular components, and yellow indicates enriched biological pathways. **(E)** Disease Ontology (DO) analysis; yellow nodes indicate the type of disease the differentially expressed PRGs are enriched in, and gray nodes indicate the genes enriched to different diseases. **(F)** Rap1 signaling pathway. **(G)** Rheumatoid arthritis pathway visualization results. Each node indicates a gene that plays an important role in the pathway, and the color of the node is determined by the log2 fold change (FC), with green indicating differentially downregulated genes and red indicating differentially upregulated genes.

We obtained 1398 differentially expressed genes in the AD group from analysis of the combined dataset (Figure 3A), 31 of which intersected with the PRGs, including 25 upregulated genes and 6 downregulated genes (Figure 3B). GO enrichment analysis showed that the differentially expressed PRGs in AD were mainly enriched in positive regulation of defense response, positive regulation of response to external stimulus, regulation of inflammatory process, and other biological processes; inflammasome complex, secretory granule membrane, secretory granule lumen as cellular components, and vascular endothelial growth factor receptor molecular function (Table 2). PRGs were linked to biological pathways such as the Rap1 signaling pathway (Figures 3D, F), rheumatoid arthritis (Figures 3D, G), and others, according to KEGG analysis. The PRGs were mostly associated with periodontal disease, bacterial infectious illness, and lung disease based on Disease Ontology enrichment (Figure 3E).

**TABLE 2:** GO and KEGG enrichment analysis results of PREDGs.

ONTOLOGY	ID	Description	P-value	q value
BP	GO:0050727	Regulation of inflammatory response	4.3701E-10	4.1631E-07
BP	GO:0032103	Positive regulation of response to external stimulus	2.5777E-08	1.2278E-05
BP	GO:0031349	Positive regulation of defense response	3.6163E-07	0.00011483
CC	GO:0061702	Inflammasome complex	0.00036483	0.02162214
CC	GO:0030667	Secretory granule membrane	0.00141337	0.02162214
CC	GO:0034774	Secretory granule lumen	0.00158686	0.02162214
MF	GO:0070851	Growth factor receptor binding	7.322E-05	0.00547222
MF	GO:0005172	Vascular endothelial growth factor receptor binding	0.00023078	0.00662694
MF	GO:0046965	Nuclear retinoid X receptor binding	0.00026601	0.00662694
KEGG	hsa05323	Rheumatoid arthritis	0.00188382	0.08307365
KEGG	hsa04015	Rap1 signaling pathway	0.00216219	0.08307365
BP	GO:0050729	Positive regulation of inflammatory response	3.6817E-06	0.00087682
BP	GO:0050673	Epithelial cell proliferation	6.1995E-06	0.0011227
BP	GO:0051090	Regulation of DNA-binding transcription factor activity	7.0712E-06	0.0011227
BP	GO:0032602	Chemokine production	1.9606E-05	0.00264818
BP	GO:0002573	Myeloid leukocyte differentiation	2.2239E-05	0.00264818

GO, Gene Ontology; BP, biological process; CC, cellular component; MF, molecular function; KEGG, Kyoto Encyclopedia of Genes and Genomes; PRDEGs, Pyroptosis-related differentially expressed genes.

### 3.2 Immune infiltration correlation analysis

**FIGURE 4:**
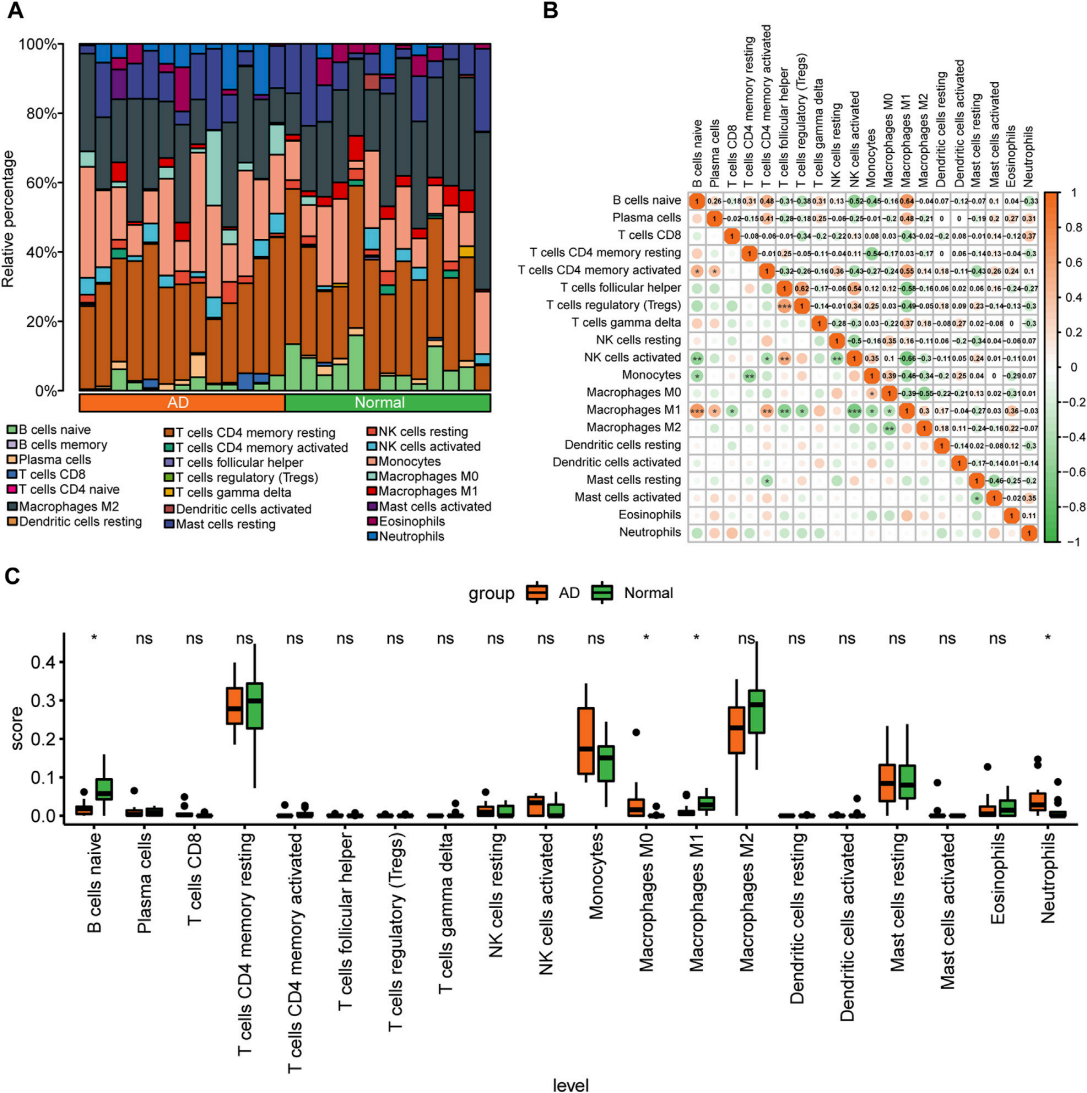
Analysis of immune cell infiltration in the combined aortic dissection (AD) dataset of GSE153434 and GSE107844. **(A)** Bar graphs of 22 immune cell types stacked in different samples of the combined dataset, with different colored long bars representing different immune cells. **(B)** Correlation analysis between various immune cells in the dataset. The color represents the correlation strength, with darker orange indicating a stronger correlation. **(C)** Differences in the abundance of enrichment of 20 immune cells in the dataset; green indicates the control group, orange indicates the AD group, the horizontal axis indicates the 20 immune cells, and the vertical axis indicates the abundance of immune cell infiltration. (ns: *p* > 0.05, *: *p* < 0.05, **: *p* < 0.01, ***: *p* < 0.001, ****: *p* < 0.0001).

The degree of infiltration of 22 immune cell types in different samples of the combined dataset is presented as a stacked bar graph in Figure 4A. Correlation analysis of infiltrating immune cells in AD revealed that M1 macrophages had a significant positive correlation with CD4^+^ memory activated T cells (*r* = 0.55), but had a significant positive correlation with naïve B cells (*r* = 0.64) and follicular helper T cells (*r* = −0.58) (Figure 4B). After removing immune cells with an immune abundance of zero (memory B cells and naïve CD4^+^ T cells), the Wilcoxon test showed that four immune cell types, including M0 macrophages, had significantly different infiltration levels between the control and AD groups from the combined dataset (*p* < 0.05). Neutrophils had considerably greater infiltration levels in the AD group, whereas B cells and M1 macrophages had significantly reduced infiltration levels in the AD group (Figure 4C), suggesting that these immune cells may play a key role in AD.

### 3.3 Gene set enrichment analysis (GSEA)

**FIGURE 5:**
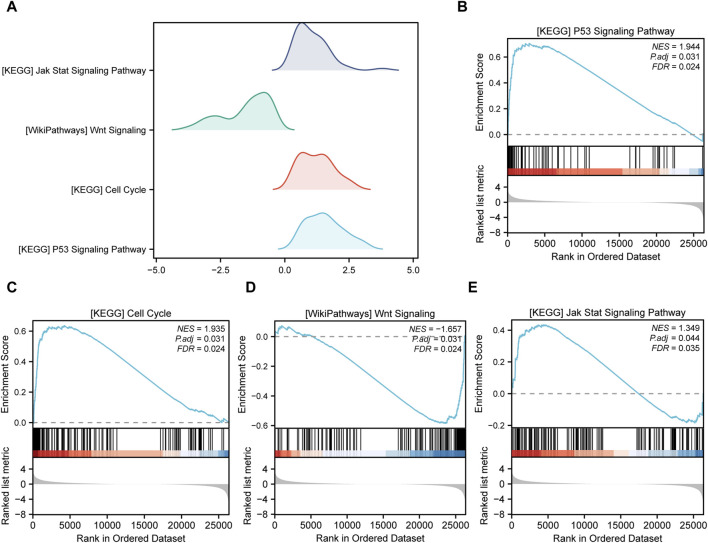
Gene set enrichment analysis (GSEA). **(A)** Four main biological features of GSEA of genes among different subgroups in the aortic dissection (AD) group. The vertical coordinate is the gene set name. **(B–E)** Genes in the AD group were significantly enriched in the KEGG p53 signaling pathway **(B)**, KEGG cell cycle **(C)**, WikiPathways Wnt signaling **(D)**, and KEGG JAK-STAT signaling pathway **(E)**. Significant enrichment was assessed at p.adjust < 0.05 and FDR value (q.value) < 0.05.

Gene Set Enrichment Analysis (GSEA) is conducted to elucidate the primary signaling pathways involved in Alzheimer’s Disease (AD). As depicted in Figure 5A, significant enrichment and upregulation (Normalized Enrichment Score = 1.944, nominal *p*-value = 0.031) of the KEGG pathway p53 signaling pathway are observed, indicating a strong overall association between apoptotic cell death and Thoracic Aortic Aneurysm and Dissection (TAAD). Furthermore, GSEA reveals significant enrichment of gene expression related to the cell cycle (Figure 5C), JAK-STAT signaling pathway (Figure 5E), and other crucial physiological pathways in AD (Table 3). Conversely, pathways such as Wnt signaling (Figure 5D) are downregulated in the AD group.

**TABLE 3:** GSEA of differentially expressed genes.

ID	NES	p.adjust	q value
KEGG_P53_SIGNALING_PATHWAY	1.94370564	0.03059695	0.02434096
KEGG_CELL_CYCLE	1.93467922	0.03059695	0.02434096
WP_WNT_SIGNALING	−1.65667156	0.03059695	0.02434096
KEGG_JAK_STAT_SIGNALING_PATHWAY	1.3491518	0.04410437	0.03508659
ROSTY_CERVICAL_CANCER_PROLIFERATION_CLUSTER	2.49282735	0.03059695	0.02434096
SOTIRIOU_BREAST_CANCER_GRADE_1_VS_3_UP	2.44015177	0.03059695	0.02434096
KOBAYASHI_EGFR_SIGNALING_24HR_DN	2.36438409	0.03059695	0.02434096
WHITEFORD_PEDIATRIC_CANCER_MARKERS	2.32871816	0.03059695	0.02434096
BLANCO_MELO_BRONCHIAL_EPITHELIAL_CELLS_INFLUENZA_A_DEL_NS1_INFECTION_DN	2.30704348	0.03059695	0.02434096
CROONQUIST_IL6_DEPRIVATION_DN	2.28660457	0.03059695	0.02434096
KONG_E2F3_TARGETS	2.27610136	0.03059695	0.02434096

GSEA, gene set enrichment analysis.

### 3.4 Screening of key genes

**FIGURE 6:**
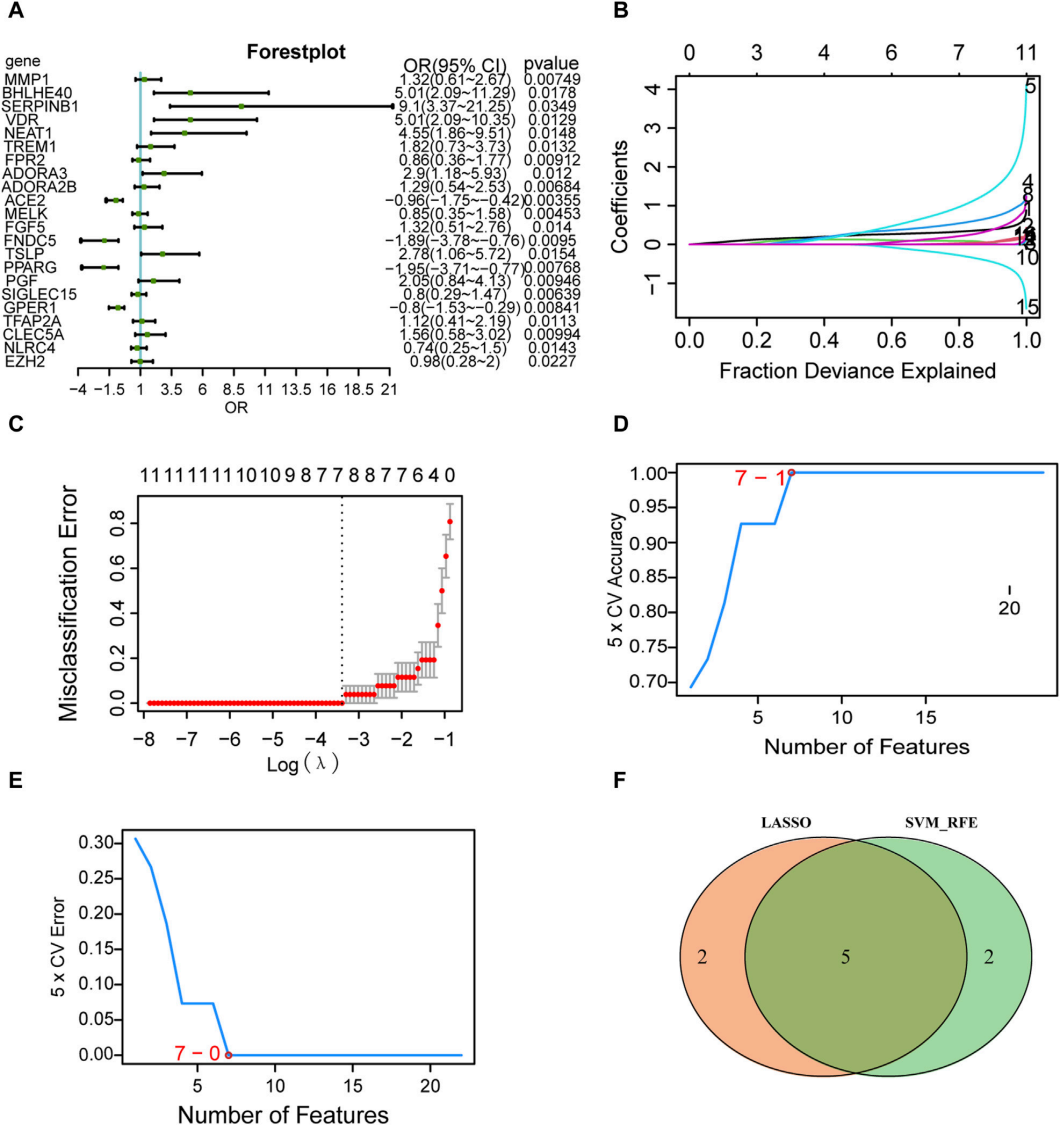
Screening of key genes. **(A)** Single-factor logistic regression forest plot of pyroptosis-related genes from the merged dataset. **(B)** LASSO regression analysis. For each gene regression covariate, positive numbers are positively correlated, and negative numbers are negatively correlated. **(C)** Vertical coordinates are the evaluation index corresponding to each λ value, and the best covariate (λ) is selected. **(D)** Number of genes with the highest accuracy obtained by the SVM algorithm. ^©^ Number of genes with the lowest error rate obtained by the SVM algorithm. **(E)** The minimum number of genes with the lowest error rate obtained by the E.SVM algorithm. **(F)** Intersection of the genes screened by the SVM algorithm (green) and the genes screened by LASSO (orange). LASSO, least absolute shrinkage and selection operator; SVM, support vector machines.

Logistic regression of the 31 differentially expressed PRGs in AD is presented as a forest plot in Figure 6A. A total of 22 genes were screened as key genes (*p* < 0.05), including matrix metallopeptidase 1 (*MMP1*), basic helix-loop-helix family member E40 (*BHLHE40*), serpin family B member 1 (*SERPINB1*), vitamin D receptor (*VDR*), nuclear paraspeckle assembly transcript 1 (*NEAT1*), triggering receptor expressed on myeloid cells 1 (*TREM1*), formyl peptide receptor 2 (*FPR2*), and adenosine A3 receptor (*ADORA3*). The LASSO logistic regression algorithm was used to identify seven genes from the 22-gene set that could be used as diagnostic markers for disease control grouping (Figures 6B, C), and the SVM-RFE algorithm was used to classify these seven genes (Figures 6D, E). The gene markers generated by the two algorithms were then superimposed to yield five diagnostic marker genes (*MMP1*, *BHLHE40*, *NEAT1*, *ADORA3*, *PPARG*; Figure 6F) as candidate essential genes in AD for further investigation.

### 3.5 Analysis of key genes

**FIGURE 7:**
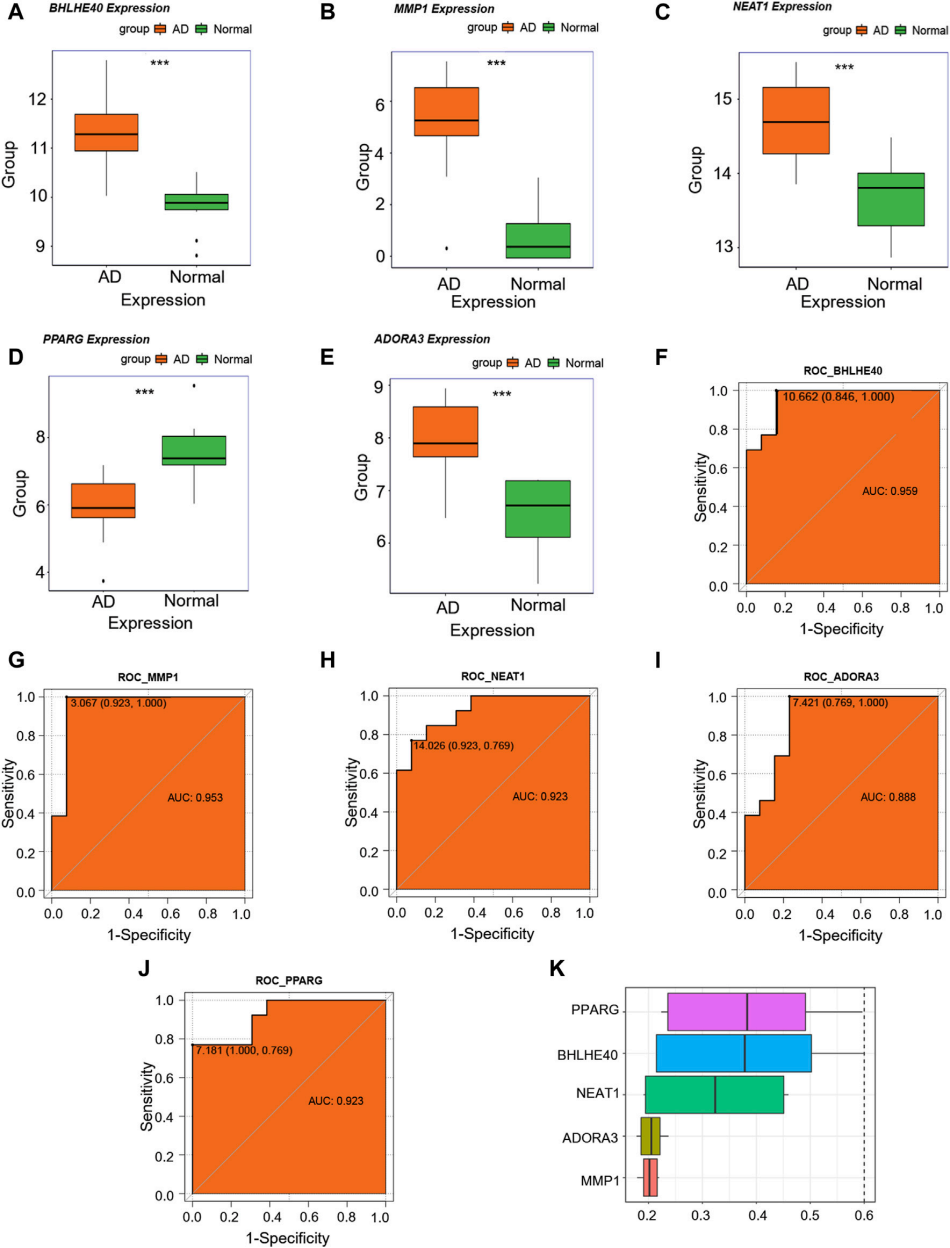
Key genes analysis. **(A–E)** Differences in expression levels of *BHLHE40* **(A)**, *MMP1* **(B)**, *NEAT1* **(C)**, *PPARG* **(D)**, and *ADORA3* ^©^ in the control (green) and aortic dissection (AD, orange). **(F–J)** Receiver operating characteristic (ROC) curves for *BHLHE40* **(F)**, *MMP1* **(G)**, *NEAT1* **(H)**, *ADORA3* **(I)**, and *PPARG* **(J)**. The horizontal coordinate is 1—specificity and the vertical coordinate is sensitivity. AUC, area under the curve. **(K)** Functional similarity analysis of key genes. (ns: *p* > 0.05, *: *p* < 0.05, **: *p* < 0.01, ***: *p* < 0.001, ****: *p* < 0.0001); the closer the AUC of the ROC curve is to 1, the better the diagnosis prediction (AUC of 0.5–0.7 indicates low accuracy, AUC of 0.7–0.9 indicates moderate accuracy, and AUC > 0.9 indicates high accuracy).

We then examined changes in the expression levels of the candidate key genes associated with AD (*MMP1*, *BHLHE40*, *NEAT1*, *ADORA3*, and *PPARG*) in the combined dataset, and discovered that *MMP1*, *BHLHE40*, *NEAT1*, and *ADORA3* were all overexpressed in the AD group compared with their levels in the control group (Figures 7A–C, E), whereas *PPARG* showed reduced expression in the AD group as opposed to the control group (Figure 7D). In addition, ROC analysis showed that *MMP1* (AUC = 0.953, Figure 7G), *BHLHE40* (AUC = 0.959, Figure 7F), *NEAT1* (AUC = 0.923, Figure 7H), and *PPARG* (AUC = 0.923, Figure 7J) all have good potential to differentiate AD patients from controls, and *ADORA3* (AUC = 0.888, Figure 7I) also has moderate potential to distinguish AD, suggesting that these genes could be used as potential diagnostic markers for AD in the future.

For functional similarity of key genes (*MMP1*, *BHLHE40*, *NEAT1*, *ADORA3*, *PPARG*), we assessed the semantic similarity of GO terms, sets of GO terms, gene products, and gene clusters using the R package GOSemSim. Figure 7K shows a box-and-line graphic of these essential genes. Among the five critical genes, *PPARG* exhibited the highest functional similarity score with other key genes.

### 3.6 Profile of WGCNA analysis

**FIGURE 8:**
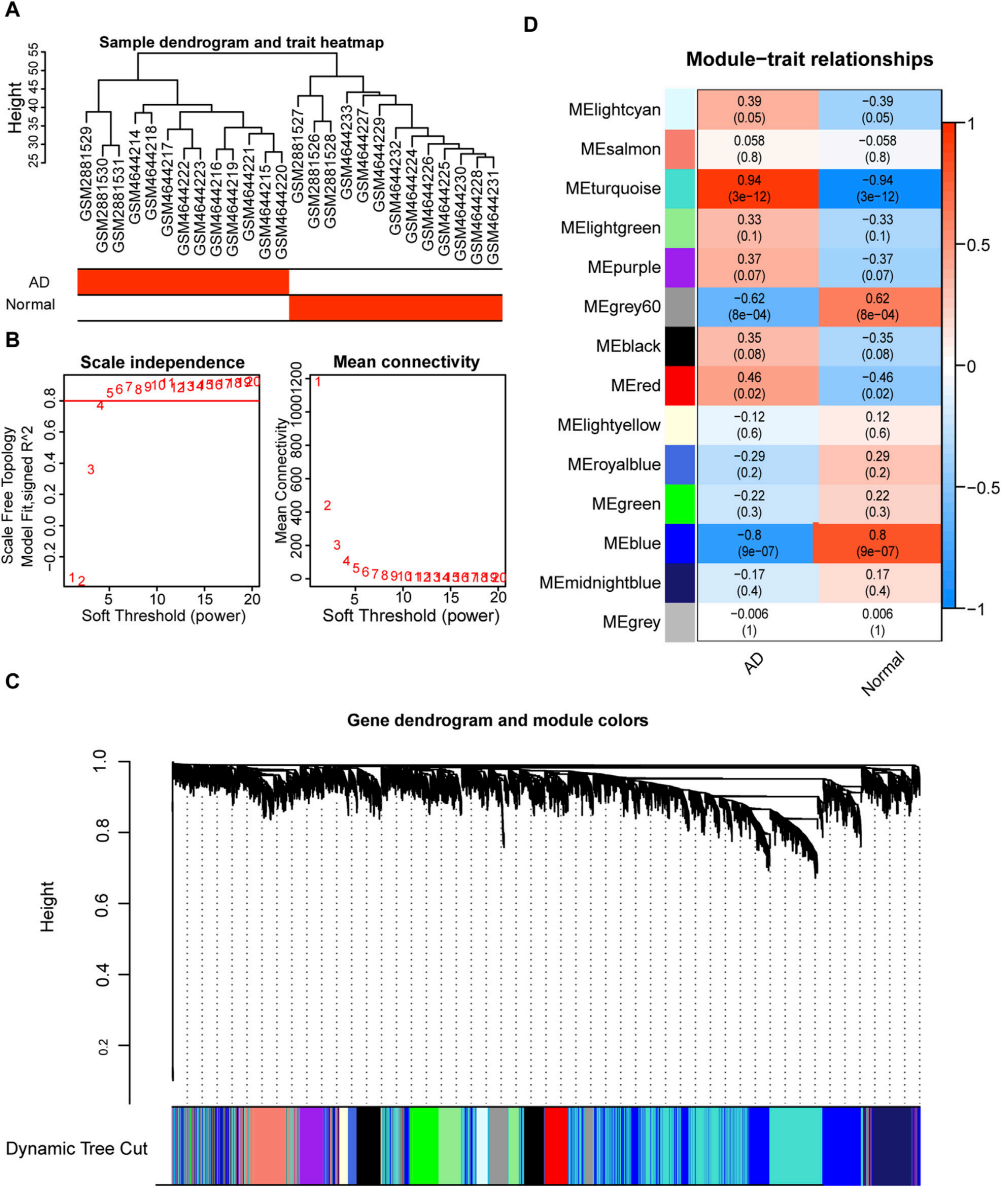
Weighted gene correlation network analysis (WGCNA) based on the expression spectrum dataset. **(A)** Sample clustering to detect abnormal samples; each branch indicates one sample and the red box in the sample information below indicates the category to which the sample belongs. **(B)** Scale-free topological model fit (left) and average connectivity (right) used to determine the best soft threshold. The horizontal coordinates of the left panel indicate the fitted soft threshold and the vertical coordinates indicate the level of fit (*R* ^2^) in the scale-free topological model. The right panel horizontal coordinate indicates the fitted soft threshold and the vertical coordinate indicates the average connectivity between modules. **(C)** Dynamic shear clustering tree for different genes. The upper tree shows gene co-expression; each gene is represented by a leaf in the tree, and each module is represented by a trunk branch. The lower colored bars indicate the corresponding seven modules and are labeled with the indicated colors. **(D)** Heat map of correlations between different modules and aortic dissection (AD) patient groups. The x-axis is the sample classification, and the y-axis is the module color name. Red indicates a positive correlation, and dark blue color indicates a negative correlation. Color blocks indicate the Pearson correlation coefficient in the upper part and the *p*-value in the lower part.

To investigate the co-expression of genes, we constructed co-expression modules using the top 25% of the genes in the combined dataset mean expression profile using the R package WGCNA. The data were hierarchically grouped using the average technique in conjunction with the patients’ clinical information (Figure 8A). The soft threshold was set to 5 to create a scale-free network (Figure 8B), with *R* ^2^ > 0.80 and good network connection. Ultimately, we identified 14 gene co-expression modules using the dynamic shear tree algorithm with each module represented by a distinct hue (Figure 8C). Correlation analysis of various modules with the AD group (Figure 8D) revealed that the turquoise module was most strongly and significantly positively correlated with the AD group (*r* = 0.94), which was therefore selected for further analysis.

### 3.7 PPI network

**FIGURE 9:**
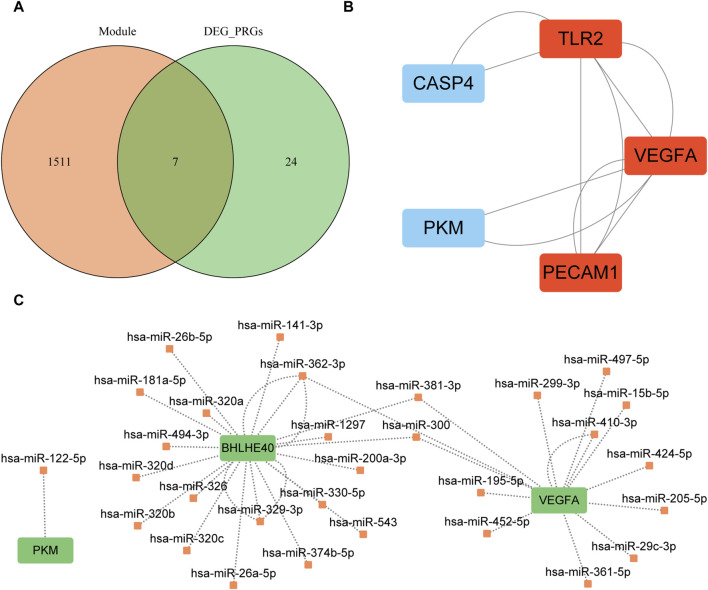
Protein–protein interaction network analysis based on seven key genes. **(A)** Intersection of the turquoise module and differentially expressed pyroptosis-related genes; orange circles indicate genes in the turquoise module, and green circles indicate differentially expressed pyroptosis-related genes. **(B)** Key modules identified by MCODE plugin of Cytoscape software; red indicates the key modules found by MCODE. **(C)** mRNA–miRNA interaction network constructed based on starBase v3.0 database prediction and visualized by Cytoscape software. Green indicates key genes and orange indicates miRNAs. MCODE, Molecular Complex Detection; PRGs, pyroptosis-related genes; DEG, differentially expression gene.

We intersected the genes in the turquoise module of WGCNA, which was closely related to the AD group, with the differentially expressed PRGs to obtain seven candidate hub genes for further analysis: *BHLHE40*, caspase 4 (*CASP4*), platelet and endothelial cell adhesion molecule 1 (*PECAM1*), pyruvate kinase M1/2 (*PKM*), serpin family B member 1 (*SERPINB1*), Toll-like receptor 2 (*TLR2*), and vascular endothelial growth factor A (*VEGFA*) (Figure 9A). Construction of the PPI network of these seven genes with the STRING database and Cytoscape (minimum required interaction score: medium confidence = 0.400; Figure 9B) revealed interactions among five proteins (CASP4, PECAM1, PKM, TLR2, VEGFA). The MCODE plugin identified a critical module of the PPI network comprising three proteins (PECAM1, TLR2, and VEGFA), as depicted in red in Figure 9B.

We used mRNA–miRNA data from the starBase database to predict miRNAs interacting with the seven hub genes (*BHLHE40*, *CASP4*, *PECAM1*, *PKM*, *SERPINB1*, *TLR2*, *VEGFA*) retaining only reliable mRNA–miRNA data pairs, and the interaction network was visualized with Cytoscape (Figure 9C). Our mRNA–miRNA interaction network included three hub genes (*BHLHE40, PKM VEGFA*), 30 miRNA molecules, and 38 pairs of mRNA–miRNA interaction connections (see Supplementary Table S2).

### 3.8 Immune infiltration correlation

**FIGURE 10:**
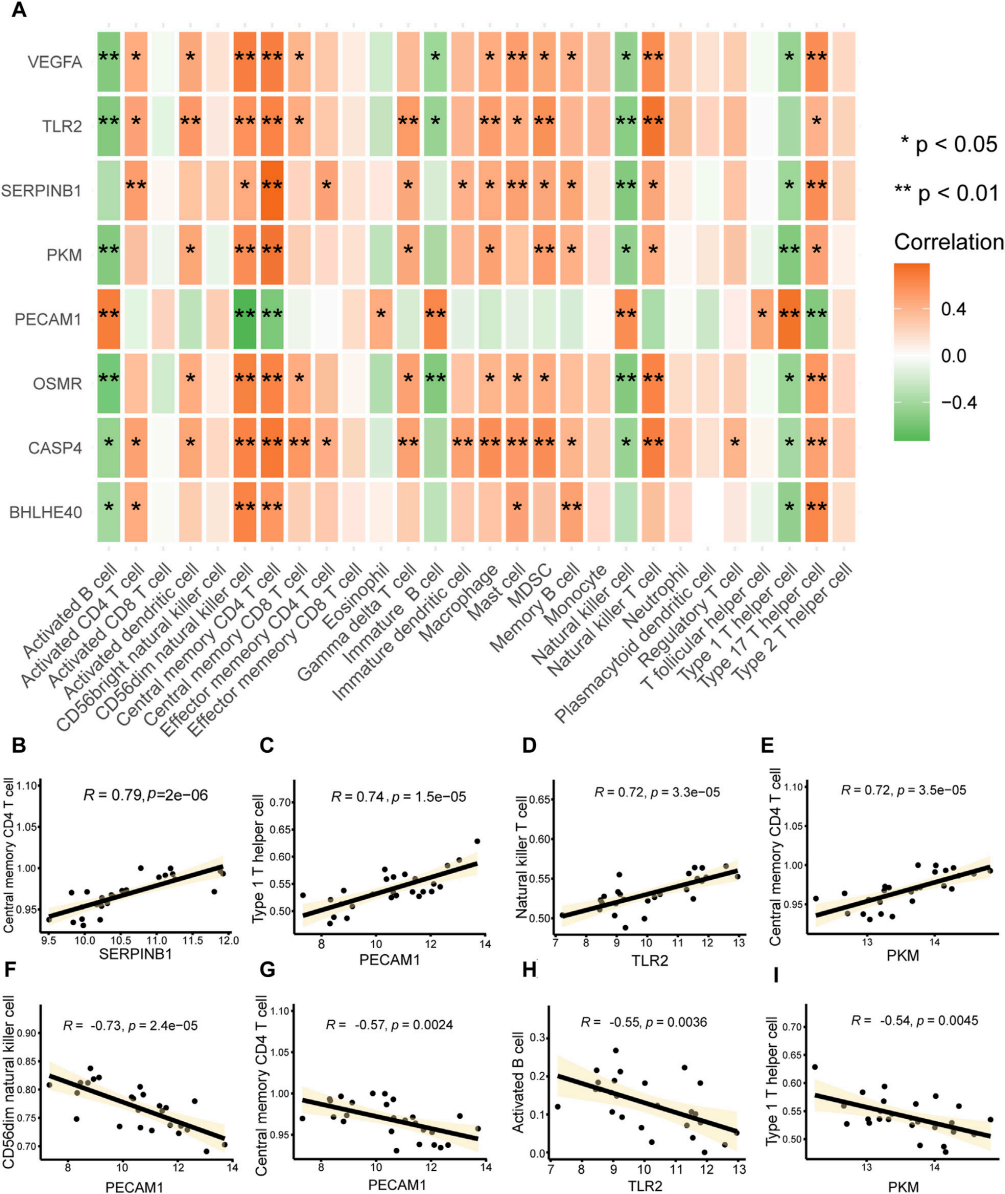
Immune infiltration correlations. **(A)** Heat map of the correlation analysis between key genes associated with pyroptosis in aortic dissection (AD) (vertical axis) and infiltrating immune cells (horizontal axis); a redder color indicates a stronger correlation between the gene and immune cells. *p < 0.05, **p < 0.01. **(B–I)** Scatter plot of the correlations between **(B)** *SERPINB1* and central memory CD4 T cells, **(E)** *PKM* and central memory CD4 T cells, **(C)** *PECAM1* and type 1 T helper cells, **(D)** *TLR2* and natural killer T cells, **(E)** *PKM* and central memory CD4 T cells, **(F)** *PCAM1* and CD56^dim^ natural killer cells, **(G)** *PCAM1* and central memory CD4 T cells, **(H)** *TLR2* and activated B cells, and **(I)** *PKM* and type 1 T helper cells. The absolute values of correlation coefficients (*r*) in the correlation scatter plots above 0.8 indicate a strong correlation; *r* = 0.5–0.8 indicates a moderate correlation; *r* = 0.3–0.5 indicates a weak correlation; and *r* < 0.3 indicates a weak or no correlation.

We computed the correlations between major PRGs and infiltrating immune cells in AD to investigate the immunological microenvironment in AD. The connections between important (hub) PRGs (*BHLHE40*, *CASP4*, *PKM*, *SERPINB1*, *TLR2*, *VEGFA*) and numerous invading immune cells in AD were generally comparable; however, the correlations between *PECAM1* expression and multiple immune infiltrating cells differed from those of the other genes (Figure 10A). There was a significant positive correlation between the expression of *SERPINB1* (*r* = 0.79, Figure 10B) and *PKM* (*r* = 0.72, Figure 10E) and central memory CD4^+^ T cells, *PECAM1* (*r* = 0.74, Figure 10C) had a significant positive correlation with type 1 T helper cells, and *TLR2* (*r* = 0.74, Figure 10D) had a significant positive correlation with natural killer T cells. Furthermore, there was a significant negative correlation between *PECAM1* and CD56^dim^ natural killer cells (*r* = −0.73, Figure 10F) and central memory CD4^+^ T cells (*r* = −0.57, Figure 10G), between *TLR2* and activated B cells (*r* = −0.55, Figure 10H), and between *PKM* and type 1 T helper cells (*r* = −0.54, Figure 10I). These results suggest that CD56^dim^ natural killer cells, central memory CD4^+^ T cells, activated B cells, type 1 T helper cells, and natural killer T cells play a significant role in the emergence of AD.

### 3.9 Drug prediction and molecular Docking

**FIGURE 11:**
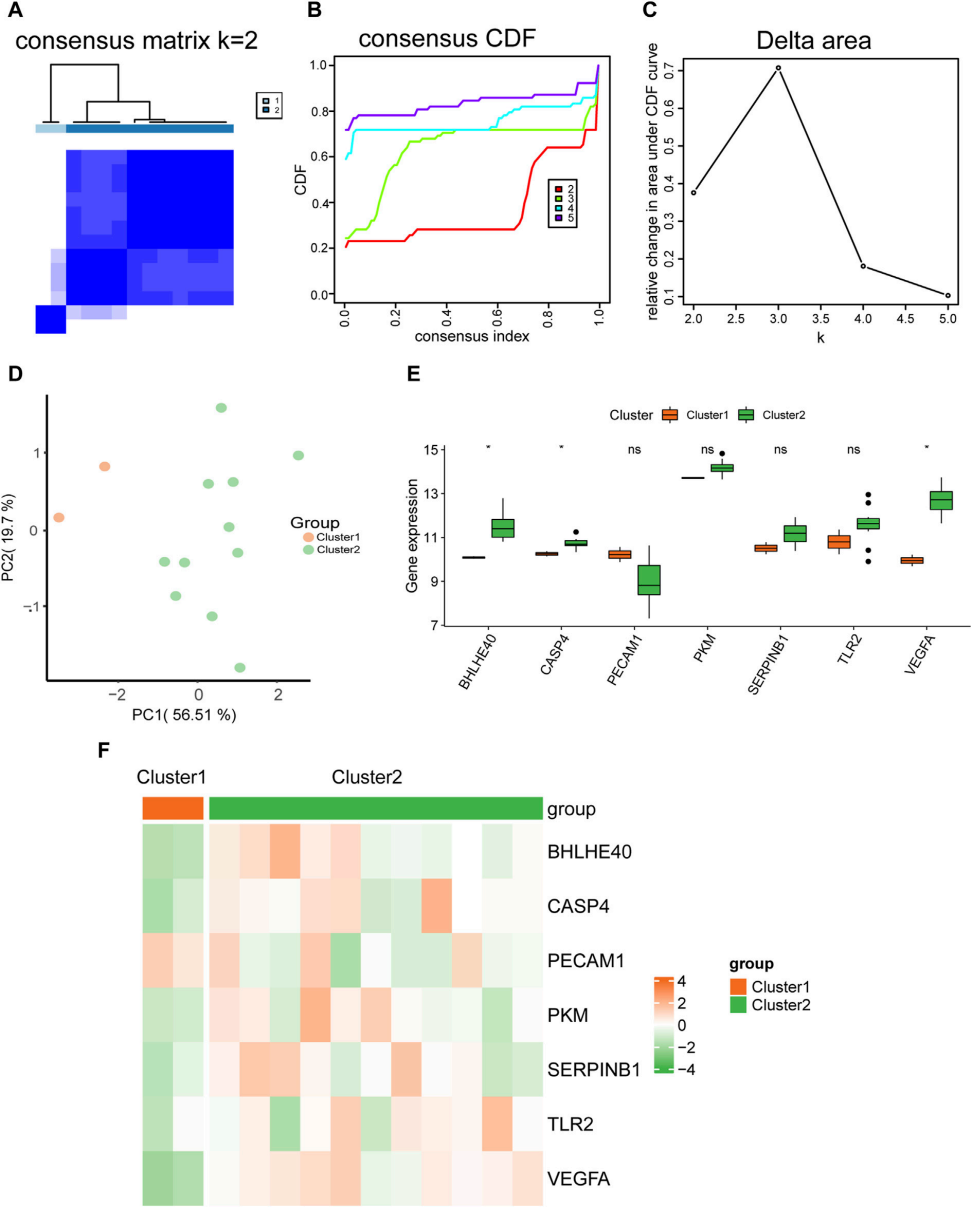
Molecular typing. **(A)** Plots of consistent clustering (K = 2) for the aortic dissection (AD) dataset. **(B,C)** Plots of the cumulative distribution function (CDF) for consistent clustering **(B)** and the area under the CDF curve delta plot for different numbers of clusters in consistent clustering **(C)**. **(D)** Principal component analysis for two AD subtypes (cluster 1 and cluster 2) in the AD dataset. **(E)** Comparison graph of the grouping of hub genes in different AD subtypes. **(F)** Heat map of the expression of hub genes in different AD subtypes. ns, not significant (*p* ≥ 0.05); *p < 0.05, **p < 0.01, ***p < 0.001.

We used expression datasets of hub genes related to pyroptosis in AD (*BHLHE40*, *CASP4*, *PECAM1*, *PKM*, *SERPINB1*, *TLR2*, *VEGFA*) to categorize patients using into subtypes using unsupervised consistent clustering. In cluster 1, including two AD samples, and cluster 2, including 11 AD samples, we could distinguish between two distinct subtypes (Figure 11A). The cumulative distribution function (CDF) plot (Figure 11B) and the area under the CDF curve delta plot (Figure 11C) demonstrate that the best consistency clustering results for the AD dataset were attained when using k = 2 as the number of clusters for unsupervised clustering. Principal component analysis on the dataset expression matrix showed that the two subtype samples of the AD dataset could be clearly distinguished from each other (Figure 11D).

We also analyzed the differences in expression of the seven hub genes (*BHLHE40*, *CASP4*, *PECAM1*, *PKM*, *SERPINB1*, *TLR2*, *VEGFA*) between the two AD subtypes (cluster 1 and cluster 2) using the Wilcoxon rank sum test. Grouped comparison plots presented in Figure 11E show that the expression levels of *BHLHE40, CASP4*, and *VEGFA* were all significantly different between the two AD subtypes (cluster 1 and cluster 2), with higher expression in AD subtype 2 (cluster 2) than in AD subtype 1 (cluster 1). The heat maps in Figure 11F further demonstrate significant differences in expression of *BHLHE40*, *CASP4*, and *VEGFA* between the two AD subtypes.

### 3.10 Analysis of hub genes

**FIGURE 12:**
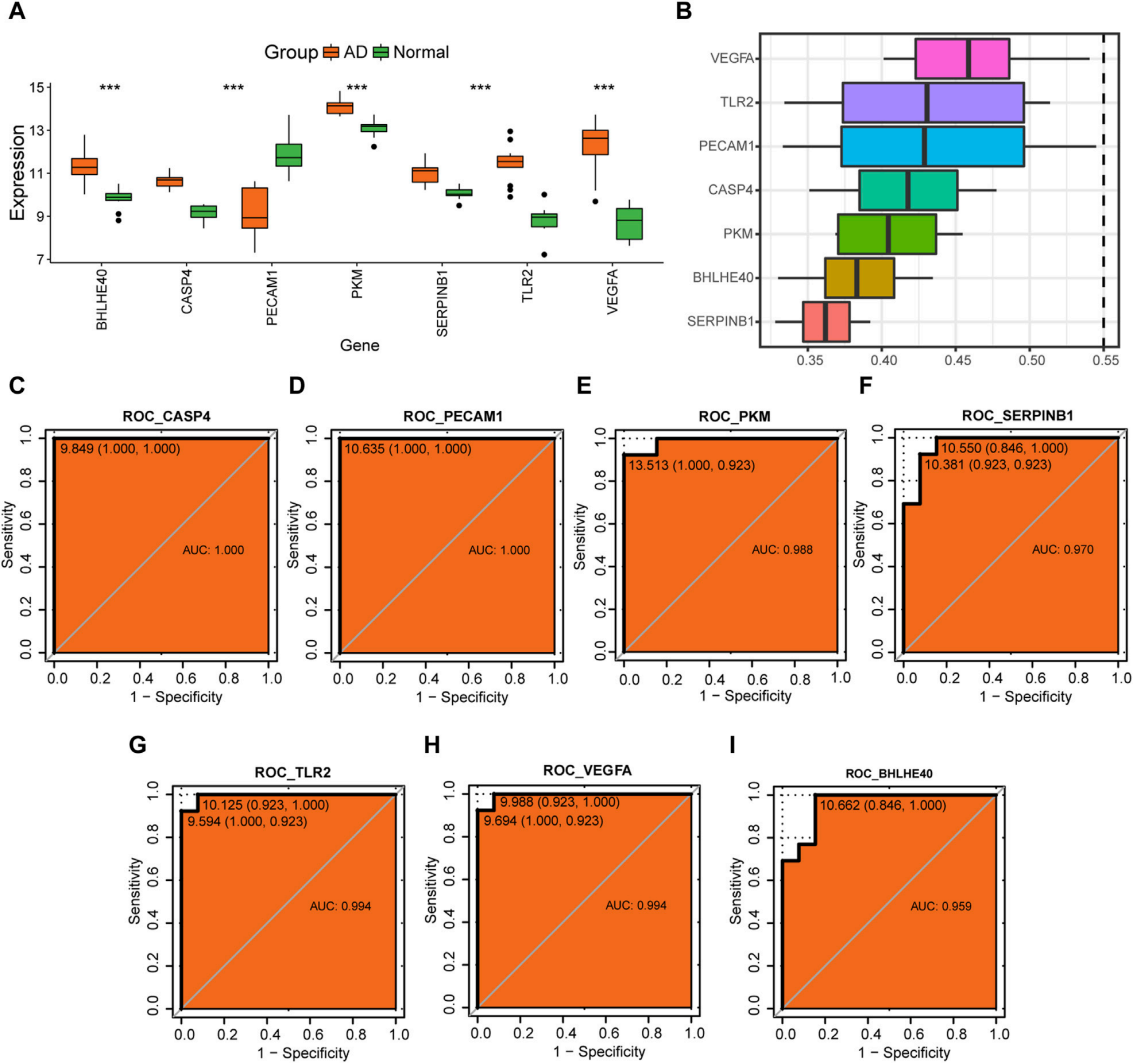
Analysis of hub genes. **(A)** Differences in expression levels of *BHLHE40*, *CASP4*, *PECAM1*, *PKM*, *SERPINB1*, *TLR2*, and *VEGFA* in the control (green) and aortic dissection (AD, orange) groups. **(B)** Functional similarity analysis of key genes. **(C–I)** Receiver operating characteristic (ROC) curves for *CASP4* **(C)**, *PECAM1* **(D)**, *PKM* **(E)**, *SERPINB1* **(F)**, *TLR2* **(G)**, *VEGFA* **(H)**, and *BHLHE40* **(I)**. The horizontal coordinate is 1—specificity and the vertical coordinate is sensitivity; AUC, area under the curve, with an AUC value closer to 1 indicating better diagnostic prediction ability. (ns: *p* > 0.05, *p < 0.05, **p < 0.01, ***p < 0.001, ****p < 0.0001).

Further analysis of the changes in expression levels of hub genes (*BHLHE40*, *CASP4*, *PECAM1*, *PKM*, *SERPINB1*, *TLR2*, *VEGFA*) associated with pyroptosis in AD in the combined dataset revealed that *BHLHE40*, *CASP4*, *PKM*, *SERPINB1*, *TLR2*, and *VEGFA* were all more highly expressed in the AD group compared to the control group, whereas *PECAM1* had a lower expression level in the AD group compared to the control group (Figure 12A). We calculated the semantic similarity between GO terms, sets of GO terms, gene products, and gene clusters of the hub genes using the GOSemSim R package for functional similarity analysis. The box line diagram of these genes is shown in Figure 12B, demonstrating that *VEGFA* had the highest functional similarity value with other key genes among the seven key genes.

ROC analysis showed that *CASP4* (AUC = 1.0, Figure 12C), *PECAM1* (AUC = 1.0, Figure 12D), *PKM* (AUC = 0.988, Figure 12E), *SERPINB1* (AUC = 0.970, Figure 12F), *TLR2* (AUC = 0.994, Figure 12G), *VEGFA* (AUC = 0.994, Figure 12H), and *BHLHE40* (AUC = 0.959, Figure 12I) all have good potential to differentiate the AD group from normal controls, suggesting that these genes could serve as potential diagnostic markers for AD in the future.

## 4 Discussion

AD is a catastrophic event caused by a tear in the aortic intima or bleeding within the aortic wall (Nienaber et al., 2016). The incidence of AD has shown an increasing trend year by year (Howard et al., 2013). Approximately 20% of the patients suffering from AD die before reaching the hospital (Golledge and Eagle, 2008). In the absence of intervention, acute AD has a 90% mortality rate (YU et al., 2020). Surgery remains the gold-standard treatment approach (Erbel et al., 2014); however, early postoperative mortality is still high, at 9%–25% (Smedberg et al., 2020). Endovascular interventions offer new treatment options for descending AD (Erbel et al., 2014); however, this approach is also associated with several possible side effects such as acute lung damage, acute renal failure, ischemia of the spinal cord, and stroke (Fattori et al., 2008). Early diagnosis and treatment of AD can dramatically enhance a patient’s prognosis. Furthermore, research into the processes contributing to the development and progression of AD can facilitate earlier detection and treatment. Recent studies have found that the hallmarks of AD include infiltration of immune cells, hyperactivation of inflammation, and degradation of the extracellular matrix, leading to vascular remodeling and weakening of the aortic wall (Lian et al., 2019). Pyroptosis and immunological infiltration have also been found to play important roles in the pathophysiology of AD. However, there is a scarcity of studies investigating the relationship between immune cell infiltration and excessive inflammatory activation in AD.

In this study, we analyzed the GSE153434 and GSE107844 datasets and performed deep data mining of the AD-related literature and public databases using bioinformatics tools with the goal of providing insight into the roles of cellular pyroptosis and immune infiltration in the development of AD and their associations. Combining datasets identified 31 PRGs that are differentially expressed in AD. GO-KEGG enrichment analyses showed that these PRGs are mainly related to the immune response, inflammasome completion, and the Rap1 signaling pathway. Biologically relevant pathways such as the p53 signaling pathway, cell cycle, and JAK-STAT signaling pathway were upregulated in AD, whereas the Wnt signaling pathway was found to be downregulated. Among the PRGs, we found that *MMP1*, *BHLHE40*, *NEAT1*, and *ADORA3* were highly expressed in the AD group, whereas the expression of *PPARG* was reduced compared with that of controls. These genes therefore have good potential to distinguish patients with AD from controls. Further analysis identified seven hub genes associated with AD (*BHLHE40*, *CASP4*, *PECAM1*, *PKM*, *SERPINB1*, *TLR2*, and *VEGFA*) that showed high diagnostic value. Among these hub PRGs, the expression of *BHLHE40*, *CASP4*, and *VEGFA* could also distinguish two AD subtypes characterized by dissection of the ascending aorta and thoracic aorta. Correlation analysis further showed enhanced infiltration of M0 macrophages and neutrophils, and decreased infiltration of naive B cells and M1 macrophages in AD samples compared with controls. The hub genes (*BHLHE40*, *CASP4*, *PKM*, *SERPINB1*, *TLR2*, *VEGFA*) were correlated with a variety of invading immune cells, suggesting the involvement of numerous immune cells, including CD56^dim^ natural killer cells, central memory CD4^+^ T cells, activated B cells, type 1 T helper cells, and natural killer T cells, in the development of AD.

Our analysis identified multiple genes with good potential to differentiate AD, suggesting their potential as biomarkers for the diagnosis of AD. Among them, *MMP1* is associated with signaling pathways of immune system cytokines that may promote persistent destruction of the aortic extracellular matrix and outer membrane degeneration (Zhang et al., 2014) to promote AD (Liao et al., 2018). *BHLHE40* encodes a transcription factor with known regulatory effects on immune cells. BHLHE40 interferes with the cytokine production of CD4^+^ T cells and with the proliferation of macrophages and CD8^+^ T cells, promotes the production of interferon-gamma by natural killer T cells (Zafar et al., 2021), increases the recruitment of neutrophils, and promotes inflammatory and hypoxic responses (Wang L. et al., 2022). We discovered a positive link between *BHLHE40* expression and a variety of immune cells, including CD4^+^ T cells, CD56^dim^ natural killer cells, type 17 T helper cells, and memory B cells, and was inversely associated with activated B cells and T follicular helper cells. *ADORA3* expression was found to be significantly increased in AD. ADORA3 has been reported to activate CD4^+^ T cells to release interleukin (IL)-10 (Durante et al., 2021), and to promote inflammatory response, apoptotic phagocytosis (Hodrea et al., 2012), and cell proliferation. *PPARG*, the only key gene that was downregulated in AD, is an anti-inflammatory gene that prevents the degeneration and death of the aortic stroma by inhibiting NF-κ-B-mediated pro-inflammatory responses (Wang M. et al., 2022) and regulates the role of the cardiovascular circadian rhythm (Aicher et al., 2021). *CASP4* is an essential pyroptotic effector that facilitates the production of IL-1 and cell death, stimulates immune cell recruitment, and activates and causes mucosal inflammation (Wang et al., 2020). We found substantial positive correlations between *CASP4* expression and the infiltration of CD4^+^ T cells, CD8^+^ T cells, macrophages, and natural killer T cells in AD. As a result, we may infer that *CASP4* plays a significant role in immune cell activation in AD and is a crucial factor in pyroptosis. Platelets, monocytes, neutrophils, and certain subtypes of T cells express PECAM1 on their surface (Dasgupta et al., 2009), which has been suggested to play a role in leukocyte motility, angiogenesis, and integrin activation, thereby inhibiting macrophage-mediated phagocytosis (Brown et al., 2002). We found substantial negative correlations between *PECAM1* expression and the infiltration of CD56^dim^ natural killer cells and central memory CD4^+^ T cells in AD. TLR2 activation encourages the upregulation of inflammatory signaling pathways, promoting T cell/natural killer T cell activation and infiltration (Moonen et al., 2019; Imanishi et al., 2020), NF-κB activation, cytokine release, and an inflammatory response (Dutta et al., 2021) and further triggers apoptosis. In addition, we discovered a substantial negative correlation between activated B cells and *TLR2* and a strong positive correlation between natural killer T cells and *TLR2*. VEGFA can induce endothelial cell proliferation, cell migration, apoptosis inhibition, and vascular permeabilization as a major contributor to both pathological and normal angiogenesis. VEGFA increases the amount of angiopoietin II and facilitates the recruitment of inflammatory cells, hence promoting inflammation (Glorioso et al., 2007). In the context of aortic dissection, there may be interactions and functional associations between VEGFA and PPARG. Regarding angiogenesis, VEGFA, as a crucial angiogenic factor, regulates angiogenesis and vascular permeability while inhibiting endothelial cell apoptosis (Melincovici et al., 2018). As a transcription factor, PPARG may modulate the expression of VEGFA (Blitek and Szymanska, 2019), thereby influencing angiogenesis and vascular permeability, further impacting the development and progression of aortic dissection. Additionally, in inflammation and immune responses, VEGFA (Chen et al., 2023) and PPAR (Toffoli et al., 2017) may mutually influence each other through the regulation of inflammation and immune responses, affecting the pathological processes of aortic dissection. Further research is warranted to deepen our understanding of their importance and mechanisms of action in this disease context. Our PPI network of key PRGs in AD showed an important module comprising interactions among PECAM1, TLR2, and VEGFA. We found a significant positive correlation between *PKM* expression and central memory CD4^+^ T cells and a significant negative correlation between *PKM* expression and type 1 T helper cells. PKM is engaged in several pathways of the innate immune system. Finally, *SERPINB1* regulates the innate immune response, inflammation, and cellular homeostasis (Choi et al., 2019) and is significantly induced in the effector CD4 cell subpopulation (Hou et al., 2019). We identified a significant positive correlation between *SERPINB1* expression and central memory CD4^+^ T cells in AD samples. Overall, our analysis demonstrated that these critical genes are involved in the control of inflammatory phenotype and immune cell activity, thereby having a significant impact on the pathogenesis and progression of AD. In particular, we found that *BHLHE40, CASP4*, and *VEGFA* can discriminate between two subtypes of AD (involvement of the thoracic or ascending aorta).

Analysis of the AD dataset demonstrated an assembly of inflammatory vesicles in AD, which stimulates the production of inflammatory cytokines as well as the death of inflammatory cells (i.e., pyroptosis) (Sharma and Kanneganti, 2021). Similarly, studies with animal models showed inflammatory vesicles in the aortic tract along with elevated serum levels of inflammatory cytokines, accompanied by pathological findings of immune infiltration, matrix degradation, and angiogenesis (Del Porto et al., 2014; Chen et al., 2022). Several signaling pathways have been implicated in the development of AD, in addition to increased inflammatory vesicles. The VEGFR signaling pathway plays an important role in aortic smooth muscle cell proliferation and migration (Wang et al., 2021). The use of VEGR tyrosine kinase inhibitors may be linked to the development of acute AD (Oshima et al., 2017; Ting and Lo, 2021). Similarly, the Rap1 signaling pathway is associated with cell migration, polarization, and proliferation (Cha et al., 2010). All of these cellular processes are implicated in the development of AD (Shah et al., 2019). Previous studies have also highlighted p53 signaling pathway-dependent proliferation inhibition, oxidative stress, and apoptosis among the potential mechanisms of AD (Wang et al., 2019; Wu et al., 2019). In addition, p53 plays a potential role in processes related to cell turnover during aortic intima-media degeneration (Ihling et al., 1999; Wang et al., 2019). The JAK-STAT pathway is extensively involved in aortic vascular injury, regulating cytokine expression and immune cell activation, and thus disease progression. This pathway stimulates the effector function of macrophages, promotes the differentiation of type 17 T helper lymphocytes, and enhances the expression of matrix metalloproteinases, ultimately leading to deterioration of the structural integrity of the vessel wall. Wnt signaling is essential for cell proliferation, differentiation, and migration. Wnt pathway inhibition enhances lipid and macrophage retention in the vessel wall as well as an increase in leukocyte-driven systemic inflammation (Borrell-pages et al., 2015). Molecules targeting the Wnt pathway attenuate intimal changes in the aorta caused by mechanical injury through the attenuation of Wnt signaling (Gay and Towler, 2017). Therefore, modulation of this common pathway might offer novel therapeutic interventions for inflammation-driven vasodilation in advanced aortic disease (Ijaz et al., 2016).

Among the multiple immune cells identified to be activated and infiltrated in AD, the infiltration of macrophages into the aortic wall is considered to be the main pathogenic mechanism of aortic injury (Guido et al., 2022). Amorphous macrophages (M0 macrophages) are driven to differentiate into a pro-inflammatory (M1) or anti-inflammatory (M2) phenotype (also known as macrophage polarization) (Xiao et al., 2020). M1 macrophages could increase and sustain the inflammatory response by secreting pro-inflammatory cytokines and promoting vascular injury (Sun et al., 2021). In addition, M0 macrophages play a key role in inflammation and immunity by recruiting more neutrophils and monocytes/macrophages and initiating further immune responses (Lian et al., 2019). The key PRGs identified in our study, including *BHLHE40*, *MMP1*, *ADORA3*, *CASP4*, and *PECAM1*, were closely associated with macrophage activation and infiltration. The levels of infiltration of M0 macrophages and neutrophil were considerably higher, whereas those of naïve B cells and M1 macrophages M1 infiltration levels were significantly lower in AD samples than in control samples, suggesting that neutrophil infiltration may dominate the acute phase of human AD. Consistent with this finding, previous studies showed that AD occurs when the expression of the outer membrane CXCL1/granulocyte colony-stimulating factor drives local neutrophil recruitment, activation, and infiltration (Yoshida et al., 2019), resulting in outer membrane inflammation, aortic dilatation, and rupture via IL-6 production (Anzai et al., 2015). We discovered a close correlation between the hub genes and immune cell infiltration, suggesting that CD56^dim^ natural killer cells, central memory CD4^+^ T cells, activated B cells, type 1 T helper cells, and natural killer T cell immunity may play an important role in the development of AD.

However, this study has certain limitations to address. Despite performing a multifaceted bioinformatics analysis, further validation studies are needed to support the present findings, including *in vivo* and *ex vivo* investigations and potential clinical trials. In addition, we have not discovered the particular mechanism of action by which pyroptosis and immune cell infiltration contribute to AD; thus, more research is needed to understand the detailed process.

In conclusion, we discovered that pyroptosis and immune cell infiltration, along with their interactions, play crucial roles in the development of AD. Changes in the expression of genes associated with pyroptosis may encourage the infiltration of immune cells in various stages of AD development, whereas the pyroptosis of immune cells may result in the strong release of inflammatory factors, thereby exacerbating the damage in various regions of the aorta. *MMP1*, *BHLHE40*, *NEAT1*, *PPARG*, *ADORA3*, *CASP4*, and *VEGFA* may be useful diagnostic markers to distinguish AD from normal controls and facilitate early detection and intervention. Among these key genes, *CASP4* and *VEGFA* could further distinguish between the two subtypes of AD. Overall, we expect that further research into the mechanisms of AD pyroptosis or immune cell infiltration will open up new avenues for the development of molecular targeted treatments for AD.

## Impact statement

Aortic dissection (AD) is a life-threatening disorder that frequently leads in mortality. Recent research suggests that pyroptosis and immune cell infiltration play a role in the pathophysiology of AD; however, the precise relationship and molecular mechanisms mediating the links between immune cell infiltration and excessive inflammatory activation in AD remain unknown. In-depth studies into the mechanisms of AD pyroptosis and immune cell infiltration will pave the way for molecularly targeted AD treatment.

## Data Availability

The datasets presented in this study can be found in online repositories. The names of the repository/repositories and accession number(s) can be found in the article/Supplementary Material.
